# Preceptors’ and nursing students’ experiences of using peer learning in primary healthcare settings: a qualitative study

**DOI:** 10.1186/s12912-022-00844-y

**Published:** 2022-03-22

**Authors:** Taghrid Jassim, Elisabeth Carlson, Mariette Bengtsson

**Affiliations:** Department of Care Science, Faculty of Health and Society, Malmö University, 205 06 Malmö, SE Sweden

**Keywords:** Learning environment, Peer learning, Physical environment, Primary health care, Structured learning activities

## Abstract

**Background:**

Due to the need for students to integrate theory with practice, current research seeks the best learning and teaching models in primary healthcare settings. The aim of this study was to explore preceptors’ and nursing students’ experiences of using peer learning during clinical practice in primary health care.

**Methods:**

A qualitative research approach was used based on semi-structured interviews with seven preceptors and ten nursing students. The interviews were transcribed and analyzed by using content analysis based on an inductive reasoning.

**Results:**

Preceptors and students perceived peer learning as an educational model to be beneficial for learning in primary care settings. They found the model to be stimulating, challenging, and leading to development of professional identity and nursing skills. All informants were positive towards the peer learning experience, with students reporting they were seen as individuals, despite working in pairs. However, the physical environment was demanding with regards to telephone counseling issues, limited opportunities for using computers, and the use of small examination rooms.

**Conclusion:**

This study shows that, despite the complex learning environment, peer learning as an educational model appears to work well in a primary healthcare setting. However, much improvement is needed to facilitate the students’ learning process. Consequently, conditions for clinical practice and learning beneficial to both students and preceptors should be prioritized by management.

## Background

Nursing students need to learn how to reflect on their practice to effectively plan, perform, and evaluate daily nursing care. This is essential in their education to become independent professionals and to ensure safe evidence-based care of good quality [1]. The practical experience from different clinical settings is therefore vital for supporting clinical decision-making and development of the nursing profession. During students’ clinical practice, registered clinical nurses act as preceptors. Because preceptors have a responsibility to create a trustful relation with the students, they must choose different teaching techniques to fit the students’ prior knowledge or level of skills [2]. Preceptorship is a complex process and requires the preceptor’s commitment, attention, and time to support and to encourage students to be reflective, independent learners [3]. However, the clinical learning environment encompasses not only the preceptor, the preceptorship, and teaching techniques, but also everything in the students’ surroundings, including the specific healthcare setting, the staff, the patients and their families, the physical environment, and equipment. Students have communicated the clinical learning environment in primary healthcare settings to be both demanding and different from hospital settings. Primary health care is the first level of contact for individuals and families within the national health- and medical care system, and it constitutes the first element of a continuing healthcare process. For students, the learning is complex because they meet patients and family members of all ages, with different care needs, and with different illnesses. Thus, students need to become competence to be able to navigate and interact with patients and their families. The clinical environment is both stressful and fluctuating – from direct patient interactions to documentation, collaboration, and other responsibilities. A primary healthcare center is often divided into independent units with different categories of healthcare staff. During students’ placement, several preceptors are actively involved in the students’ learning process – something students may find demanding [4]. However, the constant shortage of registered nurses, and thereby experienced preceptors parallel to the increasing number of students, poses an additional challenge for precepting nursing students [4]. In order to meet these challenges, innovative thinking and new strategies are needed by introducing more student-active teaching techniques and strategies such as collaborative learning – peer learning, for example. Peer learning is an educational model where students learn in collaboration. They learn from and with each other, thereby creating both an individual and a shared knowledge development [5]. Topping [6] defined peer learning as “people from similar social groupings who are not professional teachers helping each other to learn, and learning themselves, by teaching.” The pedagogical origins of this teaching and learning strategy are based on theories that embraced the virtues of social interaction and collaboration as essential elements in the construction of knowledge [7]. Thus, central to peer learning is student activity, where reflection, critical thinking, and communication are in focus to support development of clinical reasoning skills [8]. To facilitate these skills, structured learning activities (SLA) can be used as educational support for students. These are written instructions that provide guidance for students on how to collaborate while solving clinical problems. The activities are aligned to course curricula and learning outcomes to ensure that learning activities do not become random, depending on the interest and competence of a specific preceptor [9]. Stenberg et al. [9] recommend that SLA are developed in collaboration between universities and the clinical settings where students participate in clinical practice to further support student activity. Previous studies performed in hospital settings have shown that peer learning and the use of SLA can be beneficial for learning, for example leadership skills [8]. The model gives students a sense of security and increase learning [8]. It also enables reflection, stimulation, and development of students’ ability for critical thinking [10]. Additionally, previous research has also shown that the preceptor assumes a new and a more passive role and stays in the background but is there to support the students. Although working with peer learning expedited preceptors’ growth both professionally and personally, they, nevertheless, sought preparatory education and knowledge in peer learning [10]. Moreover, studies have presented a number of challenges, for example, competition among students [8], and feelings of insufficiency and stress among preceptors [10]. Therefore, the introduction of peer learning and the use of SLA are essential to adequately prepare both students and preceptors for this new student-active teaching technique [9]. Though little international attention to pedagogical aspects has been given to primary health care as a clinical learning environment [11], there is a need to develop and improve preceptorship and nursing students’ learning environment. One such approach may be the introduction of peer learning as an educational model. The aim of this study was to explore preceptors’ and nursing students’ experiences of using peer learning during clinical practice in primary healthcare.

## Methods

Students in the undergraduate nursing program at Malmö University conduct clinical practice in several settings, including four weeks in primary health care in Year 2. Primary health care has a very broad commitment to all its residents: emergency care, planned care, rehabilitation, and preventive measures. By tradition, each student was designated a specific preceptor who they followed over the four-week period. However, course evaluations showed that their learning needs were not sufficiently met. Reasons for this were described as preceptors´ lack of time with students feeling left alone, and preceptors experienced difficulties building effective relationships with the students in the short time available. Therefore, in May 2017 we implemented peer learning as an educational model in primary health care in collaboration between Malmö University and The Primary Care Education Unit in southern Sweden, which helps organize the primary care placements for students. Peer learning entails nursing students to support and learn from each other while working in pairs, without the immediate influence of the preceptor. Before the start of the clinical practice period, both preceptors and students at these primary care centers were informed about how to work with the educational model peer learning and the structured learnings activities [8, 9]. In total, nine preceptors from the participating primary health care centers took an instruction course on precepting techniques according to the peer learning model. The course comprised two days of lectures and workshops, followed by two half days dedicated to discussions based on the preceptors’ new understandings and insights. Course content included development of the educational model in preceptorship, peer learning as an educational model, and students’ and preceptors’ perspectives based on peer learning as educational model. In addition, SLA [9] were developed, focusing on, for example, fundamental nursing care, medical technical issues, telephone counselling, and communication and ethical aspects (Table 1). The first author was responsible for the instruction course with the support of a team of teachers from the Department of Care Science at Malmö University. Similar content was taught to students during their first year in a campus-based course. Before commencing clinical practice in primary health care students were reminded of how to use the SLA and thus benefit from learning with and from each other without the direct interventions of the preceptor [9].

**Table 1:** Example of a Structured Learning Activity for Year 2 nursing students in Primary health care

Structured learnings activity – Telephone counselling
**Learning subject:** Communicates, assesses, plans, and co-operates with patients, relatives, and different professional groups in the care team and patients’ chain of care
**Suitable for**: Nursing students Year 2, Primary Health Care
1) *Preparation:*
Be theoretically prepared to perform telephone counselling in a primary healthcare setting, read the guidebook for questions and advice
2) *Listen to the conversation between the preceptor and the patient, take notes in order to observe the conversation technique*
•How is the conversation performed based on evidence? Are there follow-up questions: both open and directed / closed? Describe and give concrete examples •How is the conversation carried out based on the structure and in accordance with the conversation phases? •Describe the nurse’s feedback to the patient
3) *Reflect with your fellow student*
•The conversation process: How was it? How was the theory integrated into practice? •Nurse core competence: Person-centered care – Integrated in communication? In what way? Concretize •How do you think the patient experienced the conversation? •Discuss what is important to think about if the patient had communication difficulties, such as language barriers, hearing impairment, or cognitive impairment •Reflect on what could have gone wrong and what the consequences would have been •What elements do you want to discuss with the preceptor?
4) *Feedback and discussion with the preceptor*

### Participants

All primary care centers with students from Malmö University were informed about the project, and the first six primary care centers who responded were accepted for participation. The first author apprised preceptors and students of the educational model and overall project, to which they could participate voluntarily. Twelve students and nine preceptors were invited to take part in the project. However, two students and two preceptors were not able to take part in the interviews. The reasons for dropout were related to illness, workload, and staff shortages. In total, the study group consisted of seven preceptors and ten nursing students, with the former aged between 32 and 60 years old, and the latter aged between 21 and 35 years old. Most of the participants were female: all the preceptors and five of the students. The preceptors had to take part in the training course and precept students in peer learning. All consented to be interviewed upon request.

### Data collection

Data was collected through semi structured interviews based on an interview guide with open and follow up questions addressed to both students and preceptors. An interview guide for the preceptors and another for the students were used to ensure that all topics were discussed during the interview. As the first author had assessed two of the participating students, these students and their preceptors were interviewed by an external interviewer. The interviews took place between June and December 2017, and all interviews started with collecting demographic information such as age, experience, and gender. The discussion continued with an open question, where students and the respective preceptor related their experience of using peer learning as an educational model in clinical practice: “Tell me about your experiences of peer learning as an educational model in primary health care?” Follow up questions were used for clarification, for example: “What do you mean when you say that? The interviews, which ranged from 20 to 60 min, were conducted until the point where no more new information was forthcoming. All interviews were recorded and transcribed verbatim.

### Data analysis

The transcribed data of the interviews were all read and analyzed by the first author and based on an inductive reasoning, according to Bengtsson [12]. Stage 1– decontextualisation: the author read the transcribed text several times to familiarize herself with the data and to obtain the sense of the whole, that is, finding meanings units (Table 2) by denoting the constellation of sentences or paragraphs that are central and relevant to the purpose of the study. The next step constituted the open coding process, that is, reducing the number of words without losing the essence, with each identified meaning unit labeled with a code. A coding list including explanation of the codes was created. Stage 2 – recontextualisation: the original transcript was re-read and each meaning unit was highlighted. Stage 3 – categorization: categories and sub-categories were identified and checked for internally homogeneous and externally heterogeneous. Stage 4 – the compilation: the analysis and writing up process was completed, and themes were identified. Stage 3 and Stage 4 were performed by the three authors.

**Table 2:** Overview of the analyzing process

Meanings Unit	Condensed meaning unit	Sub-category	Category	Theme
The challenge has been purely related to the physical environment. It’s difficult to find a room to work in	The challenge was purely related to the physical environment It is difficult to find a room	Difficult to find physical space for learning	Physical environment	Learning environment
I had two students that were so different, but at the same time they were highly conscious about their differences. This led to one backing off when asked to do something they were comparatively more skilled at, with the intention to let the other student try. That is not always the case, and it can become a challenge for me to engage both students in their learning	Challenging to stimulate mutual learning, challenge to engage both students in their learning and students respect for each other and cooperation	Challenging to stimulate mutual learning	Preceptors competence	Prerequisites for education

### Ethical considerations

No ethical approval was required for this study according to Swedish law [13] (SFS, 2003:460) since sensitive issues such as sexual, political, or religious questions were not asked or discussed. According to Swedish Law (SFS 2003:460) and the local ethical guidelines of the university, no written consent is necessary for studies that do not explore sensitive issues (e.g., political, sexual or religious).

However, the study was conducted in accordance with the Helsinki Declaration (WMA, 2008) [14] and local ethical guidelines set by Malmö University. Prior to the study, the preceptors and students received oral information about the project in its entirety. The participants could at any point during the process withdraw from the study. All data was handled confidentially and only available to the author. Data was stored on a passworded laptop that was not connected to the Internet during transcription.

## Results

Both preceptors and students perceived peer learning as an educational model to be beneficial for learning in primary care settings. They found the model to be stimulating, challenging, and leading to development of professional identity and nursing skills. The students perceived the peer learning experience positively, relating that they felt seen as individuals, despite working in pairs. Likewise, the preceptors were positive to peer learning because it promoted the students’ ability to take initiative and responsibility, which in turn boosted their self-confidence and independence with the various work tasks. The educational model also allowed the preceptors to abandon their traditional role, thereby permitting the students to work actively and independently, and without preceptors intervening until necessary. What is more, the preceptors noted just how quickly the students became confident enough to meet patients without supervision. However, the model required that the preceptors prepared strategies and solutions to ensure the efficiency of the educational model, especially regarding to the physical environment. Furthermore, planning and structure were necessary to ensure the quality of the students’ learning and to provide individuals with equal opportunities. Three themes emerged from the data analysis: learning environment, learning process, and prerequisites for learning (Table 3). These themes represent preceptors’ and nursing students’ experience of using peer learning in primary health care and how the model supported student learning. P is the abbreviation for preceptor and NS for nursing students.

**Table 3:** Overview of themes and categories

Themes	Category
Learning environment	Physical environment Short meetings with patients with a variety of care needs Telephone counseling
Learning process	Open communication with continuous dialogue Reflecting and independently solving tasks Learning to deal with stressful situations
Prerequisites for learning	Preceptors’ competence Time to precept Management support

### Learning environment

The physical environment and short meetings with patients with a variety of care needs were challenging and affected the preceptors’ opportunities to offer preceptorship based on the peer learning model. Moreover, the physical environment influenced how peer learning could be carried out, and the preceptors had to construct creative suggestions on how the model could be staged. Therefore, high demands were placed on the preceptors, who needed to devise new strategies for the SLA to make the educational model work effectively.

### Physical environment

The learning environment at a primary health care center is characterized by the utilization of small examination rooms, including healthcare staff, patients and two students, as well as close relatives. In addition to the nursing students, other students can attend, such as a physiotherapist-, nurse assistant-, specialist nurse-, and medical students. The preceptors found the physical environment at the primary health care center challenging for the students’ learning, particularly when it concerned two students in peer learning.

“In primary health care, it is easiest to have one student, compared to the hospital setting. We have small rooms, it can be crowded when you are more than two or three, it has been a challenge.” (P 2).

In order to deal with the limited physical space in the examination rooms, the preceptors employed varied strategies. They mostly allowed one student at a time in the room to conduct the various work tasks. Thereafter, the students reconvened to reflect on they had accomplished and then discuss with the preceptor. In this way, the preceptors retained the essences of the model regarding reflection and feedback, even though the students did not perform the task together. In other cases, the preceptors left the students with the patient, but were available for the students if needed. This was possible for those tasks that the preceptor knew the students could manage independently. Informing the patients that two students who be in attendance also served to facilitate the process.

“But of course, the physical environment is challenging, and especially due to phone counseling. It is quite difficult and crowded in that room if we are three people. We have a nice lab, but even there it is crowded…. the physical environment is not the best….This means that it can create restrictions. But I think if you tell the patient before why we are three people or two students, it will not be so strange.” (P 1).

The students experienced that the preceptors’ strategies were working and pointed out that it was always the patient who should be in focus. Therefore, students were separated in certain situations, for example, in emergency situations, when there were too many people for the patient, and when the group was too large for the examination.

“We have done a lot together and then discussed afterwards about what we have done. But we have done a lot separately and then gone back to each other and reflected afterwards. So I think it has worked really well with peer learning.” (NS 1).

The preceptors also raised the patient’s perspective, which highlighted the risk of patient discomfort with too many people in the examination room. Consequently, they sought to avoid exposing patients to situations in which they might feel secondary.

“You don’t want the patient to get into an exposed situation where the patient is sitting alone on a chair in a small room and there are at least three people standing around.” (P 3).

The students in turn saw this as a challenge for peer learning. However, students always have to think, and act based on the patient and the situation. This entailed situations in which both students could not participate and others where neither student could participate.

“Well, during an emergency situation one of us students left the room, as we felt that we might be a few too many in the room. This is probably the only situation I can think of. As with all patients, we respect if someone says that they feel uncomfortable because there are too many students and staff in the room, and then you have to listen.” (NS 2).

As there were no facilities specifically suited for the students’ duties, it was difficult for them to focus on, for example, administrative tasks undisturbed. Therefore, students used their own smartphones when doing research online. Most often, the students also lacked their own functional login to the computers and the in-house system, which made it difficult for them to work independently. This also hampered the students’ ability to work efficiently and made them dependent on the preceptors, who had to use their log on credentials for the students to be able to practice documenting. This already difficult situation was exacerbated with student pairs conducting peer learning.

### Short meetings with patients with a variety of care needs

The learning environment at a primary health care center is characterized by short meetings with patients with a variety of care needs. The preceptors revealed not only the challenge to obtain information about the patient quickly, but also the breadth of the disease spectrum and the variation of care needs at the primary health care. During these short meetings, the nurse should be able to gather enough information to ascertain a patient’s nursing care needs, resources and risks, and be able to plan and prioritize nursing care interventions. Moreover, the nurse primarily focuses on triage, which in turn requires assessment and decision-making. With many patients, shorts meetings, and varying diagnosis, independent assessments and decisions are often required. The preceptors also recognized that students might be overwhelmed by many new situations where stress levels were high with many patients to deal with. However, they acknowledged that peer learning as an educational model gave the students the opportunity to work and develop collectively, thereby learning to both understand and manage these situations. The preceptors related how well the students progressed regarding meeting patients and understanding the process.

“…In addition, you constantly meet new patients. You finished with a patient within half an hour, because it was such a short visit here. And then you would immediately move on to the next patient and make another assessment. Someone comes for abdominal pain, someone for a cough, and someone for breathing problems. It is very educational, but it is in a completely different way from the hospital’s settings.” (P 4).

“Because we were two, it became faster and easier to do things ourselves, also when we were divided. This “worry or nervousness” of being alone with the patient had been greatly alleviated by the fact that we were both students with the same background and with the same experience, and we managed it ourselves. And then I also managed myself without the support.” (NS 3).

### Telephone counseling

Telephone counseling is a major part of a nurse’s duties. However, it materialized that several nurses could be engaged in answering phones and performing other duties in the same room, thus affecting the concentration of both nurses and students. Moreover, students taking calls using a double headset was difficult. As a solution, one student undertook telephone counselling, while the other performed another activity. Thereafter, they jointly reflected on and discussed their respectively experiences. A Structured Learning Activity focusing on telephone counseling was beneficial in this situation.

“It is not possible to have three people on the phone. When I had one student at a time performing phone counselling, one could write things down and then reflect with the other and the preceptor, so you can use it that way too. At the same time, the other student could participate in another activity.”(P 5).

Despite the difficulties, preceptors believed that telephone counselling was given structure. Though the students listened individually, they still reflected on proceedings jointly thereafter. Consequently, peer learning as an educational model increased the quality of their learning through shared reflection and use of Structured Learning Activities.

### Learning process

The preceptors believed that peer learning and working with SLA developed the students’ cooperative ability through constant joint reflection. Working in pairs developed their professional role in meeting patients in stressful situations. Students reported that working in pairs rapidly made them independent and confident enough to be alone with a patient.

### Open communication with continuous dialogue

The preceptors conveyed that open communication with continuous dialogue facilitated both the preceptorship process and the students’ learning and ability to communicate. When this form of communication was missing, problems and obstacles in need of solutions were created. Moreover, it lead to negative competition and cooperation difficulties, which placed a heavy burden on preceptors in terms of finding solutions. Working with SLA force the students to communicate.

“It worked very well with the last pair; they were very straight and clear with each other, and they decided to perform tasks interchangeably. They changed constantly and had a good approach. The previous pair did not have as good communication. It did not work. Rather, it became a small competitive situation instead.” (P 4).

To resolve any lack of communication between students, preceptors employed a great deal of conversation support, both individually and in pairs. However, it was important that students took responsibility for good communication and proposed solutions.

The students acknowledged that open communication between them created structure, fostered collaboration, reduced negative competition, and facilitated the whole learning process. As a result, collaboration in the different situations was both facilitated and phased naturally. This also served to allow students to plan, organize, and allocate work assignments more easily.

“The opportunity to discuss things with preceptors existed, but then we students had communicating as well; and I know I told my fellow students ´now you sit there and I do this.´ Then we could talk afterwards and explain to each other. There were no hard feelings. As long as you had straight communication, it worked very well.” (NS 2).

The students who had been challenged in the communication process revealed they developed a lot. However, strategies and support from the preceptors were required. Students faced particular challenges, for example, different approaches to a leadership role, and dealing with applicable tasks, training, and learning opportunities. Nevertheless, it was apparent that the students developed an understanding of themselves and each other, thereby leading to them being challenged and progressing collaboratively.

“Maybe not competition in that way, but it has been positive. Because I have had to learn to believe in myself and take more space. And it has helped me now because now I still have to show the front foot [to be in charge] also so that it could be assessed. It has given me an extra push to have another student who is in the same situation.” (NS 4).

### Reflecting and independently solving tasks

Due to students reflecting and discussing with each other continuously, preceptors became more secure and confident. Students were not left to their own devices; rather, they were supportive of each other, which in turn led to preceptors being utilized for issues that required their skills.

“I see that the students’ common reflections have made them more courageous in thinking and in solving problems. They have also become faster and more independent with patient contacts, allowing them to make their own assessments and then come back to me as a preceptor and discuss.” (P 6).

Furthermore, the preceptors stated that the students in peer learning developed their skills through mutual activities, observations, discussions, and reflections. For example, they mutually thought about what questions to ask, what the diagnosis might be, when to interrupt the patient, and when to ask questions. This allowed them to make informed decisions on what course of action to take.

“… But I feel that they have become more independent quickly during patient contacts, such as making their own assessments. I think you become independent when you feel safe and gain greater confidence with coping and meeting patients.” (P 4).

Having more time for collaborative reflection and discussion resulted in more extensive and deeper reflections, which also affected the ethical perspective. The students now could reflect on their actions and observations, thus making them aware of the how and why questions.

“But just with the increased reflection time, I can feel that the possibility of applying theoretical knowledge in practical situation has become greater with reflection.” (NS 3).

The students felt that they were given more time and opportunity to reflect collectively. They appreciated that they were always available to each other, which meant that they could reflect directly on the situation independent of the preceptor. The students related that working in pairs created a learning place, which entailed that they did not have to think about or participate in all the tasks performed by the preceptor.

“We could reflect a lot as well. It came naturally when we had nothing to do or when we were going to do something; that kind of reflection came a little by itself. I think that when comparing peer-learning with other reflection time and the possibility of reflection increased tremendously for otherwise you do not spend that time to talk directly.” (NS 2).

According to the students, the peer learning gave them an opportunity for rapid independence and guidance, according to the educational model. It was seen as an aid for them and for the preceptors. Moreover, working in pairs greatly alleviated the students’ sense of anxiety concerning being alone with patients, which served to promote individual independence.

“… We did not have to rely as much on our preceptor as we could rely on each other instead, this made everything easier for us and the preceptor.” (NS 5).

The students also confirmed they gained a deeper reflection in peer learning. They were able to learn and, over time, carry out work tasks and investigations, even independently. But in peer learning, students could progress a step further. Normally, they would encounter a point where the preceptor’s input was required. However, with peer learning, they were able to reflect and discuss further without preceptor intervention.

“In a way, it obviously increases independence. If you were alone, you could have taken the ECG and done one task and the other, but maybe we could still take it a step further. There is always a point when you have to go to the preceptor. However, we could instead reflect and could go back and go a step further, and then go to the preceptor.” (NS 2).

### Learning to deal with stressful situations

Preceptors noted that students in the educational model peer learning could adapt to and manage stressful situations more easily and significantly better than those students in the traditional one-to-one model, with the latter more likely to become burdened by many patients awaiting consultation. As a result, they were likely to take care of the patients quickly, though sometimes asking the preceptors to take over. In similar situations, the students working in pairs showed more confidence and less anxiety, according to the preceptors. Because of students’ development as a result of collaboration, the preceptors maintained they were able to face and manage the challenges of the future professional role.

“Many students in the one-to-one model can be stressed when many patients are waiting in the queue. And they have also said that you [preceptor] can take over. So we are close by. They have also said that they feel that it will be so stressful. However, this is not what I noted with peer learning.” (P 4).

The students found a sense of security in having a peer available when required. Stressful situations could be managed both collectively and individually, resulting in growing student self-confidence; they could also take care of patients independently. Working in pairs not only challenged but also heightened their collaboration and work management. For the students, another notable benefit of working with the peer learning model, compared to the one-to-one method alone with a preceptor, was conducting a nurse’s daily duties. Dealing with many patients with diverse needs in a time-sensitive environment was somewhat easier with the peer learning model.

### Prerequisites for learning

Although the preceptors viewed peer learning positively, they found it challenging, nevertheless. Students communicated that they expected the preceptors to be well-trained for their roles, with the preceptors adding that they had developed in their roles. According to the latter, precepting while using peer learning as an educational model was fun, rewarding and cultivating. However, it was also time-consuming, which lead to feelings of inadequacy, stress, and frustration. Therefore, they argued for more management support and understanding, and more time for their roles.

### Development of preceptors’ competence

The preceptors maintained that education and preparation had made it easier for them to understand the essence of the peer learning model and how to apply it. Sharing experiences and discussing opportunities and obstacles during the education had been rewarding and supportive, according to the preceptors. Though they found peer learning a challenge regarding getting to know, support and motivate pairs of students, it became less complicated the more they precepted. Moreover, having two students resulted in them being more independent.

“I think it was good that I had the opportunity for education before, so that I can use the activities and understand the purpose of it. It would probably have been more difficult if I had not received some education and background about it before. I honestly do not know if I could have done it.” (P 1).

The students related a sense of security knowing the preceptor was familiar with the model. They described their preceptors as talented, knowledgeable, and supportive.

“Wouldn’t have liked it any other way. Now it certainly depends on how the preceptor is too. But it seems that our preceptor were very informed about the model itself, and she had probably planned it well before.” (NS 1).

### Time to precept

Preceptors found having two students in peer learning, as opposed to one, as more time consuming, which resulted in feelings of inadequacy, stress, and frustration. With the additional student, even more meticulous planning time was required. This was particularly time-consuming at the onset of the preceptorship period, when the preceptor needed to get to know the students, and get an idea of their learning needs and their reliability. This is the phase dedicated to preparing the students for participating at the units whenever their preceptor was not present. Limited time also affected the preceptors’ ability to reflect with the students.

“I believe that´s the biggest challenge: time and actual understanding, absolutely.” (P 2).

There was a lack of understanding amongst colleagues about how the model actually worked and preceptor guidance requirements. This could, for instance, concern explaining that certain tasks might require more time to actually execute, and time to explain and reflect with the students. The preceptors did not have the right form of support from colleagues and felt they were not able to utilize their allotted time adequately. Thus, irritated colleagues could leave the preceptor feeling deficient towards both students and workmates.

“... I wish more time and space could be given the preceptor to support the students and reflect with them. For example, I have been interrupted when I was reflecting with the students at the end of the day. I was interrupted and told to do things, even though others were available and could perform the task.” (P 4).

Moreover, the students saw that the preceptors had a heavy workload and could not devote enough time to precepting. They noticed that the preceptors did all they could to give them time for a proper introduction, reflection, and support in several forms, yet the preceptors themselves did not get the adequate support.

“It became obvious there that it was expected that the preceptor would work as usual with no consideration that she precepted two students as well. It was also clear that the planning of the days did not take this into account.” (NS 2).

### Management support

As support from managers varied between the different units, some preceptors felt that they did not get enough support and understanding. Even those preceptors that declared they had support from the management desired more, and in another forms. Requests for adequate preparation time for students prior to their four-week placement in a health center was not meet by all managers. Consequently, some preceptors had to make do with meeting the students one hour per week for reflection and feedback, plus time for mid- and final evaluation meetings. In addition, preceptors were often not permitted to attend what they deemed to be relevant courses, although it could be question of just a half day.

“I have a very good manager who is aware of the importance of having students, but I still believe we don´t really have enough time. This can cause irritation between colleagues. That´s when I wish I had some sort of support from the management. Maybe having an additional resource during the first week when the workload is at its peak. Understanding from the managers of this phase is important in order to offer the right support.” (P 4).

Students also had mixed experience of the levels of management support during the implementation phase of peer learning. A number have, clearly and early in the clinical placemen period, understood the lack of support for the preceptors and the educational model. They noted that the preceptor was not completely familiar with how peer learning would work, which in turn resulted in the preceptor reverting to the traditional model quite early in the clinical placement period.

“…and then, at the same time, there has been a bit of a split between our preceptor and her manager. Because the manager thinks that our preceptor has been away a lot on education.,. And the manager says that she [the preceptor] spends a lot of time on this with peer learning, without really giving much results so far.” (NS 6).

## Discussion

Our study exposes one unique feature of peer learning in clinical practice in this specific primary care context, namely the adaptation of the model to the conditions of the physical environment. The physical environment plays a major part in students’ learning also in clinical practice, and the limited physical space due to the number and size of rooms was addressed as one major challenge. Nevertheless, most of the participating preceptors and students had a positive experience of peer learning; and they believed that despite limitations of the physical environment, peer learning can be suitable as an educational model in a primary healthcare setting. However, the preceptors were required to find solutions by challenging their own traditional ways of preceptorship due to the educational model of peer learning. In other words, the preceptors were required to rethink and set up a plan and structure for the various learning activities. This is in line with Carlson et al. [3], who describe preceptorship as a pedagogical process that includes planning, level adaptation, application of precepting strategies, evaluation, and assessment. The preceptors allowed the students to meet patients and their families individually when the size of the examination room did not permit them to be in pairs. At the same time, the preceptors clarified the importance of and the requirements for students’ joint reflection after the activity had taken place, as well as their feedback and discussion with the preceptor. An increased and in-depth reflection between the students facilitated by clear instructions on how to reflect and what to focus on supported the students’ learning process. This is in line with Stenberg et al. [8], who report how peer learning gave the students the opportunity to collaborate and to reflect daily, as reflection was a formalized part of the structure in peer learning. The preceptors in our study tried to create a safe and structured learning environment to enable students’ opportunities for an increased sense of participation. Through this strategy, the students obtained the necessary space and time for learning both individually and in pairs [15]. Flott and Linden [16] also stated that effectiveness in facilitating learning had an impact on the outcome achievements. They also emphasized the importance of the preceptor’s role in guiding the students in applying theory to practice, in being a positive role model, and in providing constructive feedback for development [16]. The clinical learning environment should promote learning, support the application of theory to practice, and aid students in becoming proficient providers [17, 18].

Furthermore, our study revealed that preceptors actively tried to find solutions through different strategies for students to practice different activities, including telephone counseling, even though the physical environment was demanding. Most of the students and preceptors also seemed to find telephone counseling as a rewarding activity, even though this learning activity is usually overlooked and regarded as unsuitable for nursing students [19]. One reason for not letting students practice telephone counselling is that it is difficult to have two students in the telephone counselling room, where there are usually several nurses answering the phones simultaneously. Further, from a technical standpoint you can only connect one headset to each telephone, which means that only one student at a time can be with the preceptor. Nevertheless, the obstacles need to be overcome, and preceptors need to integrate innovative ways to introduce and engage students in telephone counselling [19], since this activity is a rapidly growing area. Telephone counseling is thus something specific to primary care, and it is precisely in this clinical placement that students can practice and learn about it. It is therefore important to take advantage of the peer learning model and the interaction and collaboration between students in relation to telephone counseling. In addition to the demanding physical environment, communication with patients by telephone is also a challenge to teach. Telenursing, unlike other traditional ways of care, offers recommendations and advice to the persons seeking care without visual references [20]. Students need some kind of guidance, but assessments skills used in face-to-face consultations are not directly transferable to telephone counseling [21].

Telephone counselling can be thereby be implemented as a SLA with guidance on how students can take turns either listening to the nurse providing telephone counselling or observing the actions that take place during the session in what Markowski et al. [22] refer to as scripted peer observation including reflection together before and after the activity.

We argue that one major benefit is that the opportunity to listen to an experienced nurse leads to increased reflection between the students, and thereby a deeper understanding of a nurse’s role in providing health advice. These conversations provide students with learning opportunities in different health areas and include various aspects, such as self-care, ethical perspectives and ethical dilemmas, assessment of care needs, and priorities. Additionally, solving SLAs without the preceptor being present permitted students to work independently and was experienced as time saving for the preceptor, which is consistent with the recent study by Stenberg et al. [9]. Conclusively, we suggest that problem-based activities like SLAs with built-in structured and mandatory reflection between students probably promote a scientific approach to knowledge in both theory and practice, and we recommend such activities as a pedagogical tools during clinical placements. The results of the current study also show that the students developed their communicative ability and the ability to reflect and collaborate. This is in line with Lister and associates [23], who argue that incorporating telenursing into education will help to provide a more confident and adaptive nurse. Nevertheless, the preceptors struggled to find time for reflection with the students. This is in line with Carlson et al.’s [24] argument that the main challenge for a majority of preceptors seems to be lack of time for preceptorship.

Additionally, in contrast to the preceptors, the students did not consider the physical environment problematic. The students pointed out that it is always the patient who should be in focus, and therefore, it was obvious for the students to be separated when needed. Students and preceptors conveyed that the structure that peer learning offers had been useful to introduce ways of collaborative learning when the limitation of physical space did not let them meet patients in pairs or take active part in, for example, telephone counselling. Brammer [25] highlights the importance of the learning environment having direct and indirect impact on students’ learning and ways in which students interact with others. Learning is, consequently, an ongoing external interaction between students and learning environments. Preceptors’ strategies and attitudes can affect whether this interaction goes well or not. In the current study, it can be perceived that the preceptors’ strategies to handle the problem facilitated and created conditions for the students’ learning in peer learning. This is also in line with Stockhousen [26] and Solvoll [27], who confirm that student-preceptor interactions and discussions promote student learning, and that students reported unmet learning needs when the preceptors did not prepare them for situations. Using peer learning as an educational model in a pedagogical strategy promoted the students’ ability to take initiative, which increased the students’ self-confidence and independence. These findings where are also confirmed by Pålsson et.al [28] and Hellström [29], and may indicate that peer learning can been helpful for students to approach their upcoming professional role and to deal with stressful situations in a setting where limited time is allocated for each patient. However, preceptors need opportunities to prepare for their role and how to assume responsibility for providing qualitative precepting. This is also in line with Nygren and Carlson [10], who suggest that preceptors support each other by providing collegial support as a means to discuss problematic situations continuously.

We acknowledge the risk for researcher bias as the authors have long experience as educators and clinical teaching using the peer learning model in theoretical and clinical courses. Moreover, the authors are teaching preceptors of the model, and are thereby theoretically well-grounded in the concept. On the one hand, this might be seen as a limitation. Therefore, to avoid influencing the participants during interviews and later the analysis of data, we have been aware of the need for self-reflection and continuous dialogue between the researcher until consensus has been negotiated and agreed. On the other hand, it is beneficial that the researchers have a solid knowledge of the studied field as we have been able to address both challenges and benefits during data collection and analysis [30]. Two of the authors (EC and MB) have neither been teaching nor assessing participating students nor were they acquainted to any of preceptors. However we do acknowledge that the first author (TJ) had a teacher relationship to the students as well as a collegial relationship to the preceptors which potentially might have made participants, specifically the students, obliged to be interviewed. The interviews were therefore conducted after the students had left their clinical placement with passing grades.

## Conclusions

In summary, from our point of view, and despite the complex learning environment, peer learning as a learning model seems to work well in primary health care. However, to better facilitate the students’ learning process, much improvement is needed. The students should be given priority, and the preceptorship as an important responsibility for nurses should be highlighted. We propose to systematically evaluate the learning environment and, above all, the physical learning environment in order to create space for the students: their own places and equipment for learning and training. Further, the clinical environment as pedagogical place, space, and room in clinical practice is still a relatively unexplored area and renders continued interest.

## Data Availability

The datasets generated and/or analyzed during the current study are not publicly available due No data available due to Swedish ethical regulation but are available from the corresponding author on reasonable request.

## Abbreviation

SLA: Structured learning activity
